# *Ex Vivo* Interaction Between Human Gut Microbiota and Artemisinin: A Multi-Omics Perspective

**DOI:** 10.1021/acsomega.5c01983

**Published:** 2025-05-15

**Authors:** Paulo Wender Portal Gomes, Helena Mannochio-Russo, Stanislav N. lablokov, Dmitry A. Rodionov, Heejung Yang, Pieter C. Dorrestein, Scott N. Peterson, Christine Tara Peterson

**Affiliations:** † Faculty of Chemistry, Institute of Exact and Natural Sciences, Federal University of Pará, Belém, PA 66075-110, Brazil; ‡ Collaborative Mass Spectrometry Innovation Center, Skaggs School of Pharmacy and Pharmaceutical Sciences, 8784 University of California San Diego, La Jolla, California 92093, United States; § Phenobiome Inc., Palm Springs, California 92262, United States; ∥ Sanford Burnham Prebys Medical Discovery Institute, Bioinformatics and Structural Biology Program, La Jolla, California 92037, United States; ⊥ College of Pharmacy, Kangwon National University, Chuncheon 200-701, Republic of Korea; # Sanford Burnham Prebys Medical Discovery Institute, Tumor Microenvironment and Cancer Immunology Program, La Jolla, California 92037, United States; ∇ Center of Excellence for Research and Training in Integrative Health, Department of Family Medicine, University of California San Diego, School of Medicine, La Jolla, California 92093-6607, United States

## Abstract

*Artemisia* is a plant genus historically used in Ayurveda and traditional Chinese medicine for its diverse pharmacological properties. Among the many phytochemicals found in *Artemisia* species, artemisinin is notable for its antimalarial activity. This compound is commonly isolated from Artemisia annua and is known to be biotransformed by fungi such as *Aspergillus*. However, there is limited knowledge on whether the gut microbiota can mediate artemisinin’s biotransformation. To address this gap, this study explores for the first time the association between human gut microbiota and artemisinin-derived metabolites across three *Artemisia* species herbal extracts and purified artemisinin. Our findings describe the first sulfated artemisinin (C_3_H_6_NO_3_S) potentially modified by bacteria Romboutsia sedimentorum and Citrobacter freundii. Thus, we highlight how a multiomics approach to natural products can reveal interactions between *Artemisia* species and the gut microbiota. This understanding can enhance the knowledge of artemisinin metabolism in the human gut and contribute to the development of novel, effective therapeutic strategies.

## Graphical abstract

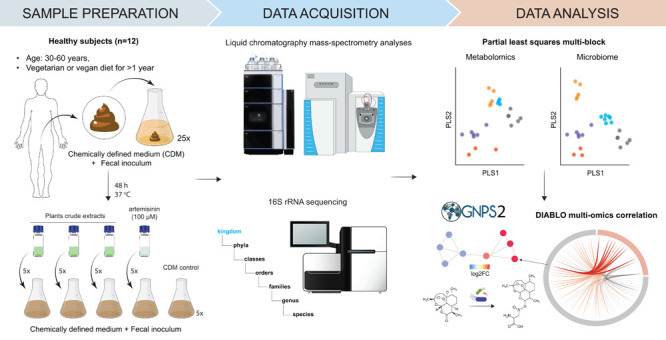

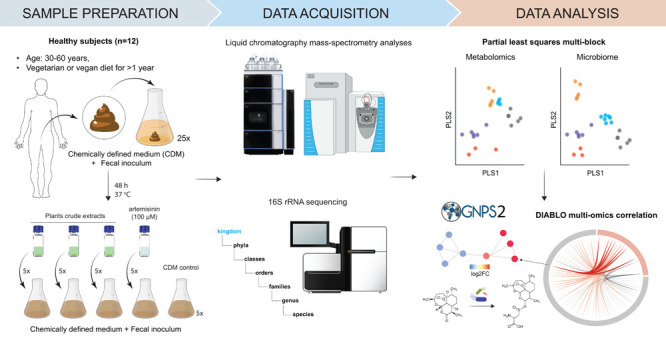

*Artemisia* is a genus with over 500 species distributed in temperate zones worldwide, and it has been used in various clinical applications. Among the species, Artemisia annua is notable for being the main producer of artemisinin—a drug used to treat malaria.^1^ Furthermore, the traditional system of medicine from India, namely Ayurveda, and traditional Chinese medicine are known for their use of many *Artemisia* species associated with numerous pharmacological properties, including neuroprotective, antifungal, antimicrobial, insecticidal, anthelmintic, hepatoprotective, and antidepressant effects.^2–6^ These activities might be associated either directly or indirectly with artemisinin. For instance, a recent study revealed that artemisinin is a promising agent for cancer therapy,^7^ and even to support immunity and treat liver conditions.^8^

Further investigation revealed that artemisinin’s anticancer mechanism is similar to its antimalarial mechanism. When reacting with iron, artemisinin forms free radicals, leading to cellular damage and apoptosis of high-iron-containing cells, such as cancer cells.^9^ Recently, extracts derived from A. annua have been reported as a phytopreparation to treat COVID-19 infections^10^ associated with systemic inflammation. Therefore, artemisinin and its derivatives commonly figure among the main potential natural products for treating several diseases.^11,12^

Considering the intricate connection between systemic inflammation and gut health, we hypothesize that the gut microbiota could interact with artemisinin-enriched extracts and, thus, may directly or indirectly promote its anti-inflammatory effects. The gut microbiota, a complex community of microorganisms residing in the digestive tract, plays a crucial role in maintaining human health as well as contributing to several diseases.^13^ Research has delved into the potential effects of various natural compounds on gut microbial composition, and *Artemisia* species have recently attracted attention for their diverse pharmacological properties, including potential impacts on the gut microbiota.^14^ Here, we propose a novel strategy for natural product discovery that takes into account the activities and metabolites of the gut microbiota to maximize efficient drug discovery. While little is known about the effects of *Artemisia* extracts or artemisinin on the gut microbiota, an animal model pilot study reported that *Artemisia* extracts increased the Bacteroidetes to Firmicutes ratio in obese murine mucosal samples and induced significantly greater microbial diversity within the mucosal compartment relative to the luminal microbiota.^15^ Thus, *Artemisia* extracts or artemisinin may, in turn, contribute to modulating immune responses and reducing inflammation.

To date, animal model studies have reported that artemisinin and its derivatives regulate glucose metabolism and remodel the gut microbiota in high-fat diet-induced mice. For instance, dihydroartemisinin—a semisynthetic derivative of artemisinin has been shown to decrease serum triglycerides, modulate gut microbiota composition, and exhibit potential therapeutic effects for hyperlipidemia, inflammation, and neurodegenerative disorders.^16^ Additionally, it has been linked to reduced depression-like behaviors in mice^17^ and exhibited strong anticolorectal cancer (CRC) activity via cell cycle arrest, apoptosis induction, and anti-inflammatory actions.^18^ Artemether, another artemisinin derivative, significantly improved gut dysbiosis caused by a high-fat, high-fructose diet and alleviated intestinal barrier dysfunction and inflammatory responses in a mouse model.^19^

Considering the findings outlined previously, it is critical to determine if extracts from *Artemisia* species or even the pure compound can be affected by the human gut microbiota. To further understand artemisinin’s broad pleiotropic effects, herein, we compare the effects of extracts from three *Artemisia* species (A. annua, Artemisia californica, and Artemisia absinthium) and isolated (or purified) artemisinin on human gut microbes. Using a multiomics approach, we also evaluate the effects on gut microbiota composition and the biotransformation of herb metabolites *ex vivo*. We hypothesize that human gut microbiota mediates the therapeutic effects of Artemisia species through plant compound metabolism and/or health-promoting microbial metabolite generation. Therefore, this study addresses the *Artemisia*-gut microbiota interactions, enhances therapeutic mechanistic understanding, and informs novel therapeutic strategy development.

## Results and Discussion

In this study, we explored the influence of gut microbiota on the biotransformation of *Artemisia*-derived constituents. This endeavor is ideally suited for *ex vivo* cultivation of fecal microbiota as it removes the complicating features of host-derived activities. Replicate cultures, capturing natural variability in herb-induced microbiota modulatory potential, facilitate correlation analysis between specific taxa and metabolites.

### Gut Microbiome

*Artemisia* species modulate fecal microbiota community structure. We inoculated human fecal material in a chemically defined medium lacking carbohydrate sources with and without supplementation of medicinal herbs or purified artemisinin. Genomic DNAs derived from these cultures were used to amplify the V3–V4 region of the 16S rRNA gene and sequenced on the Mi-Seq platform. These sequences were enumerated at multiple levels to enable approximate species-level comparisons. In total, we observed 673 unique phylotypes (ASVs) and an average of 168 phylotypes (range = 114–233) in each unique culture condition. We calculated alpha diversity using Shannon indices. Also, we calculated Bray–Curtis distances at the phylum level to create the PCoA reflecting the beta diversity of the control and herb/artemisinin-supplemented communities to determine whether structural and compositional differences in these herbs differentially alter the composition of fecal communities. We observed that A. californica and purified artemisinin-supplemented cultures did not significantly alter alpha diversity measures, whereas A. annua and A. absinthium supplementation resulted in increased alpha diversity (Figure 1a). Compared to control cultures, the beta diversity of cultures supplemented with purified artemisinin was not significantly different, whereas cultures supplemented with A. absinthium and A. californica formed discrete and well-separated clusters, and cultures supplemented with A. annua displayed the greatest modulation (Figure 1b).

**Figure 1:**
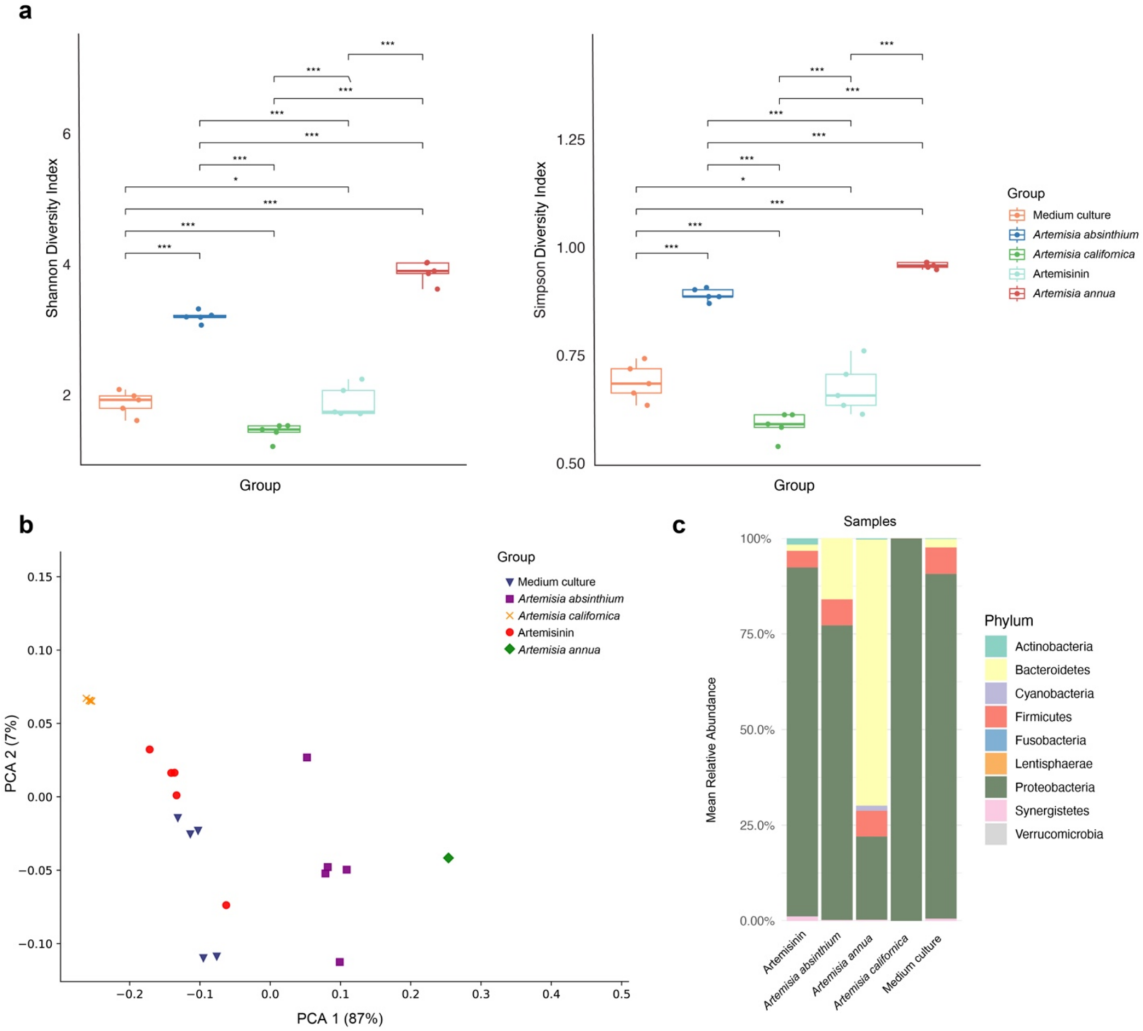
Diversity measures of control cultures compared to those supplemented with artemisinin and medicinal herbs. (a) Comparison of observed ASVs in control and herb-supplemented cultures. Increased/decreased relative abundance using a 5-fold cutoff. Shannon and Simpson’s alpha diversity measures of control cultures compared to those supplemented with medicinal herbs. (b) Principal coordinates analysis (PCA) shows the beta diversity of control, artemisinin, and medicinal herb-supplemented cultures. (c) Average relative abundance (%) of bacterial top 9 taxa in different sample groups. Each bar segment represents the proportion of a specific bacterial phylum in the total bacterial community. The Kruskal–Wallis test was used to identify if at least one group differed from the others, and then the Wilcoxon test was used for pairwise comparisons. * = *p*-value >0.05 indicates there are no underlying differences, and *** = *p*-value <0.008 indicates there are significant statistical differences.

Through alpha diversity results (Figure 1a), we observed that A. absinthium and A. annua supplementation had the most pronounced effects on microbial diversity, whereas A. californica and purified artemisinin had little effect. The results suggest that these herbal treatments provide prebiotic-like effects and indicate their capacity to promote a more varied microbial ecosystem. Increased microbial diversity is often associated with a healthier gut environment, potentially providing resilience against disease and promoting overall gut health. This interpretation is supported in part by the lack of prebiotic effects displayed by purified artemisinin supplementation, which, compared to medicinal herbs, lacks various components present in herbs such as glycans and other microbial fitness determinants. The differential impact across *Artemisia* species highlights the specificity of each herb’s influence on the gut ecosystem. In addition, the beta diversity analysis (Figure 1b) provided further insights as we observed small but clear shifts in cultures supplemented with A. californica despite its negligible impact on alpha diversity. A closer inspection of taxa impacted by A. californica indicates that it had the strongest negative impact on the relative abundance of microbial taxa. Interestingly, cultures treated with A. annua showed a marked divergence from controls. These cultures displayed greater intracultural variability. The reasons for broad differences in community composition are unclear but may reflect stochastic differences in culture replicates.

Figure 1c presents the mean relative abundance of bacterial phyla in fecal cultures treated with different *Artemisia* species and artemisinin. The control group (Medium culture) is dominated by Firmicutes and Bacteroidetes. A. annua caused the most significant shift, with a substantial decrease in Firmicutes and an increase in Bacteroidetes and Proteobacteria. A. absinthium led to a dramatic increase in Actinobacteria, becoming the dominant phylum, and a decrease in Firmicutes. A. californica showed a moderate shift with an increase in Bacteroidetes and Proteobacteria and a decrease in Firmicutes. Artemisinin had a relatively minor effect. These changes suggest potential alterations in short-chain fatty acids (SCFA) production (e.g., acetate, propionate, and butyrate), gut barrier function, and inflammatory potential.^20^ The decrease in Firmicutes may reduce butyrate production, while the increase in Bacteroidetes might enhance acetate and propionate production.^21^ The increase in Actinobacteria with A. absinthium suggests potential probiotic effects.^22^ However, the increase in Proteobacteria with A. annua *and A. californica* warrants further investigation as it could indicate dysbiosis.^20^ It is important to note that these are *ex vivo* findings, and further *in vivo* studies are needed to confirm these results.

### Integration of Metabolomics and Microbiome

Metabolomics data distribution is represented by the Principal Component Analysis (PCA) plot (Figure 2a), which reveals distinct clusters of crude extracts compared to extracts inoculated with human fecal cultures. This separation leads to significant metabolic profile variations and reflects the gut microbiome activity (biotransformation) on medicinal herb constituents. In addition, microbiome and metabolomics data were integrated using the DIABLO approach.^23^ The samples clustered into distinct groups (Figure 2b). This suggests that different treatments (e.g., different Artemisia species, the addition of fecal inoculum, or the control medium culture) have led to different metabolic and microbial profiles. Some overlap between clusters was observed, particularly for A. absinthium and A. annua, which might indicate that these two species share some common metabolic and microbial effects. Thus, the strong correlation between the metabolomic and microbiome data suggests that the microbial community plays a crucial role in shaping the metabolic profile of the samples. This relationship could have implications for understanding the mechanisms by which *Artemisia* species and microbes modify sample metabolites.

**Figure 2:**
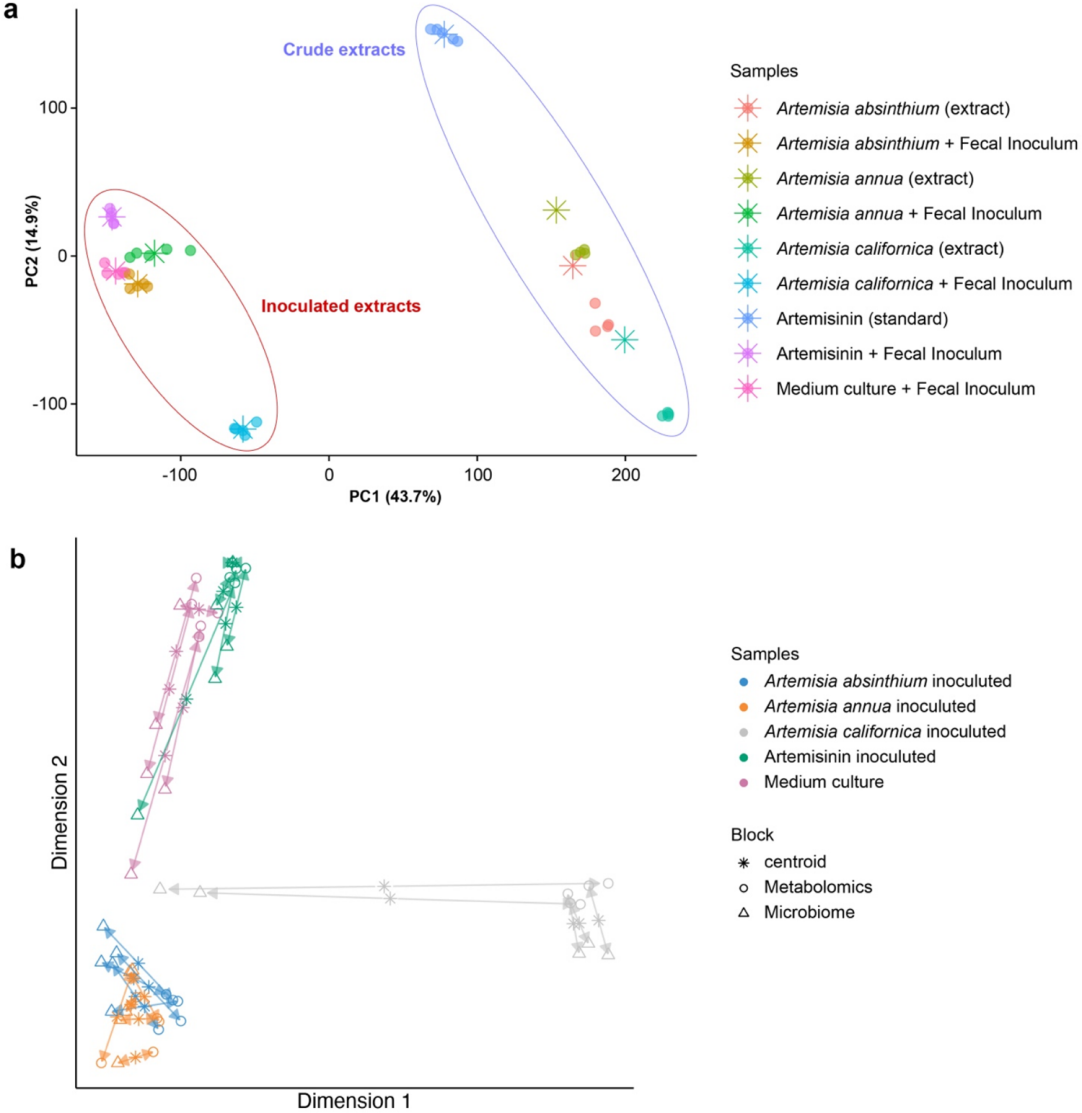
Principal component analysis (PCA) of metabolomics data, along with partial least squares (PLS) modeling that integrates metabolomics and microbiome data. This analysis compares samples of Artemisia species and artemisinin (standard) inoculated with human fecal inoculum, emphasizing distinct clustering patterns and correlations between the data sets. Panel (a) presents a PCA for the extracts from *Artemisia* species before and after the addition of the fecal culture inoculum. The PCA shows the clustering of samples based on their metabolic profiles, underscoring the effect of fecal inoculum on metabolomic variance. Dots of different colors represent the samples, while asterisks indicate the centroid for each group of samples. The PLS plot in panel (b) compares the variates of integrated metabolomic and microbiome data (both involving fecal culture). Symbols and colors distinguish sample blocks (metabolomics and microbiome). The plots reveal clear separation patterns in metabolomic and microbiome profiles as a result of fecal inoculum treatment.

The correlation based on the DIABLO approach^23^ to connect microbiome to metabolomics data shows two well-connected bacteria to an artemisinin-derived (Figure 3). The circular correlation network (Figure 3a), employing a correlation cutoff of *r* ≥ 0.65, visually represents the positive (red lines) and negative (gray lines) relationships between specific bacterial taxa and metabolites. Fortunately, in our study, we did find evidence that the *ex vivo* gut microbiota could convert artemisinin into dihydroartemisinin—a compound previously described with many health benefits. Moreover, the correlation network underscores a strong association between Romboutsia sedimentorum and Citrobacter freundii, and various artemisinin-modified molecules. It should be noted that these bacteria are phylogenetically distinct, representing both gram (−) and gram (+) microbes. The molecular network (Figure 3b) illustrates the biotransformation pathways of artemisinin and its derivatives, highlighting compounds (red nodes) with increased concentrations in fecal-inoculated extracts. It proposes a specific biotransformation of artemisinin to a molecule annotated at level 2 of the Metabolomics Standards Initiative (MSI)^24^ potentially mediated by R. sedimentorum and/or C. freundii (Figure 3c). Previous evidence confirms that C. freundii is a nontraditional sulfate-reducing bacterium,^25^ which could explain the bioconversion of artemisinin to that metabolite with the molecular formula C_3_H_6_NO_3_S, which is suggested by MS/MS spectral data to contain a cysteine sulfinic acid-derived moiety conjugated to the artemisinin core. In the gut environment, cysteine sulfinic acid may arise from dietary sources, microbial metabolism, or host metabolic processes. The gut microbiota is known to play a crucial role in sulfur metabolism, including the degradation and transformation of sulfur-containing amino acids like cysteine.^26^ Given that taurine is synthesized through cysteine sulfinic acid and taurine-conjugated metabolites are commonly observed in biological systems, it is plausible that cysteine sulfinic acid could serve as a conjugation partner for artemisinin or its derivatives. This conjugation might occur enzymatically or through nonenzymatic reactions facilitated by the gut microbiota. Figure 3d shows a mirror match between both MS/MS spectra of artemisinin (*m*/*z* 283.1549) and its derived metabolite (*m*/*z* 420.1672). The spectra reveal similar fragmentation patterns for artemisinin and its modified form, allowing us to hypothesize that *m*/*z* 420.1672 is a derived molecule likely modified by microbes. In addition, we compared the relative concentrations of artemisinin and its product (artemisinin-C_3_H_6_NO_3_S) of bacteria biotransformation across *Artemisia* extracts and artemisinin (purified) based on the raw peak areas (relative intensity/counts of ions), as shown in Figure 3e,f, respectively. In Figure 3e, the raw peak areas of artemisinin (standard) across A. absinthium, A. californica, and A. annua show a higher relative concentration in samples without fecal culture. In contrast, Figure 3f reveals that artemisinin decreased after adding fecal inoculum, while the biotransformation product showed a significant increase in all samples investigated. Thus, these results highlight significant changes in artemisinin content before and after exposure to fecal inoculum, underscoring the role of microbial activity in metabolizing artemisinin and its related compounds.

**Figure 3:**
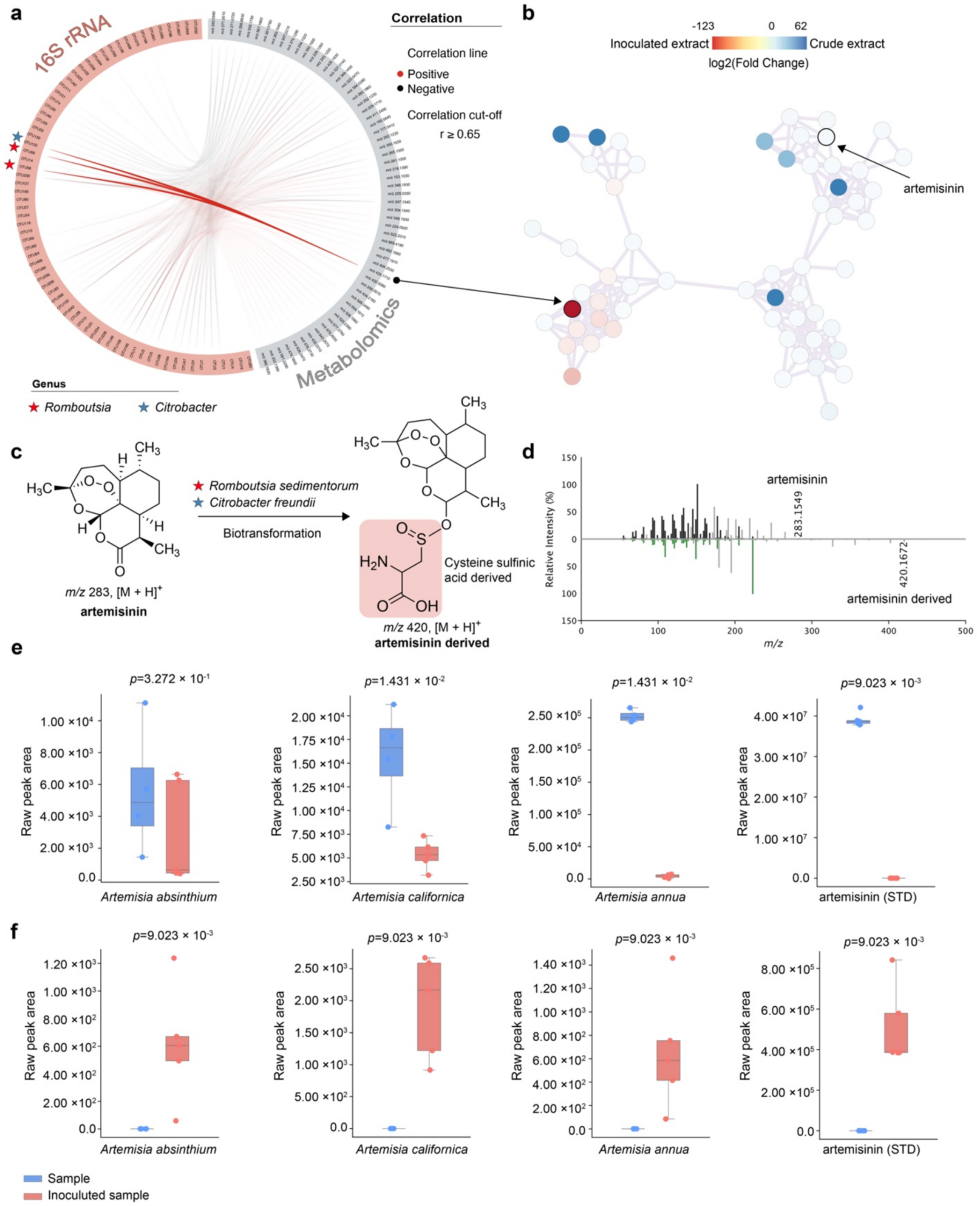
Multipanel illustration of the correlation between the microbiome and metabolites from Artemisia species, along with microbial biotransformation of artemisinin. (a) Correlation network based on the DIABLO approach,^23^ showing relationships between microbial species and metabolites. Positive correlations (*r* ≥ 0.65) are indicated by red lines and negative correlations by black lines. (b) Molecular network of artemisinin-derived metabolites, with nodes colored by log2fold change in abundance between crude and fecal-inoculated extracts. The putative artemisinin biotransformation product is marked. (c) Proposed microbial biotransformation of artemisinin into a derivative conjugated to a moiety with the molecular formula C_3_H_6_NO_3_S. (c) Mirror plot comparing MS/MS spectra of artemisinin (*m*/*z* 283.1549) and its biotransformed metabolite (*m*/*z* 420.1672), indicating structural modifications. (d) Mirror plot comparing MS/MS spectra of artemisinin (*m*/*z* 283.1549) and its biotransformed metabolite (*m*/*z* 420.1672), indicating structural modifications. The *x*-axis represents the *m*/*z* values (mass-to-charge ratio), while the *y*-axis indicates relative ion intensity. (e) Box plots showing the raw peak areas of artemisinin in A. absinthium, A. californica, A. annua, and pure artemisinin before (blue) and after (red) fecal culture inoculation. (f) Box plots depicting the raw peak areas of the artemisinin biotransformation product (artemisinin-C_3_H_6_NO_3_S) in the same conditions as panel e. Data represent five (5×) biological replicates per condition. Statistical significance across the samples was determined using the Wilcoxon signed-rank test.

Through integrating metabolomics and gut microbiome data, we observed a significant impact of fecal inoculum on the artemisinin-derived molecular profile. The introduction of fecal inoculum causes substantial changes in the overall profile of artemisinin, generating analog molecules. These changes are shown by the boxplots of artemisinin before (blue boxes) and after (red boxes) treatment (Figure 3). We associated the decrease of artemisinin due to its biotransformation intermediate by bacteria to sulfur artemisinin-derived, as such confirmed by the boxplots in Figure 3e, in which we putatively detected artemisinin-C_3_H_6_NO_3_S (*m*/*z* 450.1806). We hypothesized that the strong correlation (*r* ≥ 0.65) between artemisinin-C_3_H_6_NO_3_S and R. sedimentorum, and C. freundii (Figure 3a) could explain this biotransformation, and data from the literature suggest gut microbe sulfate compounds as a pathway for detoxification processes.^27^ Furthermore, a previous study reported artemisinin derivatives containing sulfur atoms with high selectivity and potent cytotoxicity activity against cancer cell lines.^28^ Another study reported a sulfated polysaccharide that exhibited significant antimalarial activity.^29^ We cannot affirm that artemisinin-C_3_H_6_NO_3_S possesses cytotoxic or antimalarial activity since we did not isolate this compound due to a lack of biomass. However, our findings describe for the first time a sulfated artemisinin modified by bacteria R. sedimentorum, and C. freundii. Thus, these results allow us to hypothesize future studies and highlight those changes in the microbial community composition that mirror changes in the metabolic profiles. This integrated approach highlights the complex interplay between herb metabolism and microbiome composition. Therefore, the findings described here underscore the importance of considering both metabolic and microbial aspects when studying the effects of treatments on drugs of natural products. The distinct profiles observed for different *Artemisia* species and treatments can inform further research on their specific properties and potential therapeutic uses. Additionally, the observed changes in microbiome composition could provide insights into the mechanisms underlying the effects of fecal inoculum on host metabolism.

Given the results presented here, we define a new question that may be widespread in drug therapies that should address the extent to which therapeutic effects derived from artemisinin are attributable to the parent compound and/or whether microbial-generated metabolites of drugs may provide previously unrecognized therapeutic effects. In the case of artemisinin, the activities of the gut microbiota may be negative via the reduction of artemisinin levels or positive due to the biotransformation of artemisinin to compounds with potentially higher bioactivity. Challenges in this field include the need for more rigorous and standardized research methodologies, considering variations in *Artemisia* species and individual responses. Future studies should explore the specific mechanisms through which *Artemisia* compounds interact with gut microbes, clarifying whether these interactions are species-specific or generalizable across the genus. Thus, scientific exploration of *Artemisia* species’ impact on the gut microbiota represents a burgeoning area of research with potential implications for human health. As we unravel the complexities of the gut-microbiota-artemisia nexus, a more comprehensive understanding of how natural compounds influence our internal microbial communities will undoubtedly contribute to the development of novel therapeutic strategies for gut-related and other conditions. Continued interdisciplinary research holds the key to unlocking the full therapeutic potential of *Artemisia* in the context of gut health.

### Limitations

While this study, using a multiomics approach, provides novel insights into the interaction between *Artemisia* species, artemisinin, and the *ex vivo* human gut microbiota, some limitations should be acknowledged. First, although our microbiome analyses revealed shifts in community composition and identified potential microbial taxa associated with artemisinin biotransformation, targeted bacterial culture experiments and enzymatic assays remain necessary to confirm microbial metabolism. Also, our findings indicate the presence of an artemisinin-derived metabolite with a proposed molecular formula (C_3_H_6_NO_3_S) at level 2 of annotation based on the Metabolomics Standards Initiative (MSI).^24^ Thus, future studies employing organic synthesis and high-resolution nuclear magnetic resonance (NMR) spectroscopy will be required to characterize this metabolite to confirm its chemical structure at level 1 and biological significance. As such, follow-up studies involving host-microbe interactions, immune responses, and additional metabolic pathways, gnotobiotic or humanized microbiome models could provide additional mechanistic insights. Despite these limitations, our findings represent an important step toward understanding how gut microbiota interact with *Artemisia*-derived compounds and lay the foundation for future investigations into their metabolic fate and therapeutic potential.

## Materials and Methods

### Study Participants and Sample Collection

Twelve healthy, English-speaking women and men aged 30–60 years who had previously adhered to a vegetarian or vegan diet for >1 year were recruited to donate a single stool sample. Participants ate their normal diets and donated a morning fecal sample in stool hats (Fisher Scientific). The fecal samples were transferred to conical tubes and stored at −80 °C until further processing. This study was carried out following the recommendations of the Sanford Burnham Prebys Medical Discovery Institute Institutional Review Board and guidelines. All subjects gave written, informed consent following the Declaration of Helsinki. The protocol was approved by the Sanford Burnham Prebys Medical Discovery Institute’s Institutional Review Board (IRB-2014-020).

Medicinal herbs were examined in the current microbiome study. We examined 3 medicinal herbs derived from *Artemisia* species and one isolate from A. annua, namely artemisinin. Medicinal herbs were sourced as follows: A. annua (Pacific Botanicals; Grants Pass, OR), A. californica (Tierramor; San Jose, CA), and A. absinthium (Elanen Naturals; Westlake Village, CA). Purified artemisinin was obtained from Double Wood Supplements (Philadelphia, PA).

Anaerobic fecal cultures. Healthy stool samples used for comparison were derived from an equal volume mixture of stool collected from 12 healthy vegetarian participants as a fecal pool. This fecal pool was used to inoculate a chemically defined medium supplemented with medicinal herbs (1% w/v). Purified artemisinin was used at a concentration of 100 μM. Approximately 10^6^ cells were introduced into a chemically defined medium to allow multiple doublings before achieving saturation. Cultures were grown under anaerobic conditions (9% H_2_, 10% CO_2_, 81% N_2_), statically for 2 days at 37 °C. Fecal samples (*n* = 25) from 12 healthy subjects were pooled and inoculated into a chemically defined medium (CDM). Each pooled culture was grown in technical replicates (*n* = 5) to ensure reproducibility. All cultures were grown to approximate saturation and harvested by centrifugation. The recovered material was used immediately for genomic DNA isolation.

### Culture Medium

Chemically defined medium (chemically defined medium) contains 50 mM *N*-2-hydroxyethylpiperazine-*N*’-2-ethanesulfonic acid (HEPES), 2.2 mM KH_2_PO_4_, 10 mM Na_2_HPO_4_, 60 mM NaHCO_3_, 4 mM of each amino acid except leucine (15 mM), 10 mL ATCC, Trace Mineral Supplement. The chemically defined medium contained nucleoside bases (100 mg/L), inosine, xanthine, adenine, guanine, cytosine, thymidine, and uracil (400 mg/L). The chemically defined medium contained choline (100 mg/L), ascorbic acid (500 mg/L), lipoic acid (2 mg/L), hemin (1.2 mg/L), and myo-inositol (400 mg/L). Resazurin (1 mg/L) was added to visually monitor dissolved oxygen. The pH of the media was adjusted to 7.4. Medicinal herbs (2% w/v) in sterile water and 2X chemically defined medium were prereduced separately in an anaerobic chamber (Coy Laboratories) for 2 days. Equal volumes of medicinal herb and 2X chemically defined medium were combined just before inoculation to achieve 1x chemically defined medium containing 1% medicinal herb.

### Microbial DNA Isolation

Genomic DNA was isolated from cultures as well as the fecal inoculum using the procedures of the QiaAmp DNA stool kit (Qiagen) with a modification that included an additional step of bead beating using the Thermo FastPrep instrument (MP Bio) to ensure uniform lysis of bacterial cells. DNA was purified with QIAquick (Qiagen) purification kit columns. DNA integrity was analyzed by spectrophotometry and visualized by gel electrophoresis. Quantitative PCR was used to allow equivalent amounts of each amplicon generated in each sample to be pooled for library construction.

### 16S rRNA Sequence Analysis

Multiplexed 16S rRNA libraries were prepared using standard 16S rRNA metagenomic sequencing library protocols from Illumina, which uses the V3–V4 region of 16S rRNA for target amplification and subsequent analysis. We used Qiime 2 for all other taxonomic analyses at the species level and higher and subsequent genome reconstruction. Briefly, raw sequence reads were filtered, denoised, paired-read merged, and chimeras removed using the default parameters in dada2 ^31^ to generate an abundance table with amplicon sequence variants (ASVs) representing individual 16S rRNA sequences. To assign taxonomic descriptions to the obtained ASVs, we used the multitaxonomy approach (MTA). Each ASV sequence was aligned with 16S rRNA sequences from the Ribosomal Database Project (RDP, version 11.5). The obtained alignments were sorted by the percent identity with maximum values denoted as *M*. We further collected and processed taxonomic assignments for identified 16S rRNA sequences with identities higher than the *M*–(1–*M*)/4 threshold. The resulting multitaxonomy assignments comprised one or more taxonomic names separated by “/”. To account for variable 16S rRNA gene copy numbers in reference genomes, we further renormalized each sample′s ASV abundance by average 16S rRNA copy numbers at each taxonomic level provided by the rrnDB database.^32^

### Chemicals

Acetonitrile (ACN) and water with 0.1% formic acid LC-MS grade were used as mobile phases for LC-MS analyses and were acquired from Thermo Fisher Scientific (San Diego, CA, USA). Analytical-grade chemicals were purchased from Sigma-Aldrich (Steinheim, Germany).

### Sample Preparation, Metabolomics, and Data Processing

An aliquot of 200 μL from each fecal-herb coculture supernatant was solubilized with 200 μL of methanol/H_2_O (1:1) with 1 μM of sulfadimethoxine (internal standard) and then, transferred to a C-18 SPE cartridge (previously conditioned) to clean up. The resultant solutions were transferred to a 96-well plate (2 mL each), centrifuged for 20 min at 2000 rpm and 4 °C, and dried in a speed vacuum. All dried samples were kept at −80 °C until resuspended.

The samples were resuspended in 200 μL of ACN/H_2_O (1:1) with 1 μM sulfachloropyridazine (internal standard for metabolomic analyses). Subsequently, the plates underwent sonication in an ultrasound bath (Branson 2800, Danbury, CT, USA) for 5 min, followed by vortexing for approximately 10 s, and centrifugation for 20 min at 2000 rpm and 4 °C. Aliquots of 150 μL from each sample were then transferred using a multichannel pipet to a 200-μL ThermoScientific 96-well plate for LC-MS/MS analysis. A blank composed of ACN/H_2_O (1:1) with 1 mM sulfachloropyridazine was prepared, along with a quality control (QC) mixture containing sulfamethazine (C_12_H_14_N_4_O_2_S), sulfamethizole (C_9_H_10_N_4_O_2_S_2_), sulfachloropyridazine (C_10_H_9_ClN_4_O_2_S), sulfadimethoxine (C_12_H_14_N_4_O_4_S), amitriptyline (C_20_H_23_N·HCl), and coumarin-314 (C_18_H_19_NO_4_).

Metabolomic analyses were conducted using a Vanquish UHPLC system paired with a Q-Exactive Orbitrap mass spectrometer (Thermo Fisher Scientific, Waltham, MA, USA), managed through Thermo SII for Xcalibur software (Thermo Fisher Scientific, Waltham, MA, USA). Chromatographic separation was achieved on a Kinetex C18 column (50 × 2.1 mm, 1.7 μm particle size, 100 Å pore size, Phenomenex, Torrance, CA, USA) utilizing a high-pressure binary system for gradient elution. The column and autosampler were maintained at 40 and 25 °C, respectively. The flow rate was set at 0.5 mL/min, with elution performed using ultrapure water (solvent A) and acetonitrile (solvent B), both acidified with 0.1% formic acid (FA). The gradient method was as follows: 0–0.5 min at 5% B; 0.5–8.0 min from 5 to 100% B; 8.0–11.0 min at 100% B; 11.0–12.0 min from 100% to 5% B; and 12.0–14.0 min at 5% B to stabilize the system before the next analysis.

A data-dependent acquisition (DDA) method was utilized for the mass spectrometry analysis, covering an *m*/*z* range of 80 to 2000 Da and using an electrospray ion source in positive ionization mode. External calibration was performed using a sodium formate solution (Thermo Fisher Scientific, San Diego, CA, USA), achieving an error margin of less than 0.5 ppm. The operational settings included a spray voltage of 3.5 kV, sheath nitrogen gas pressure of 35 psi, and auxiliary nitrogen gas pressure of 10 psi. The ion source temperature was maintained at 270 °C, with an S-lens RF level of 60 V, and the auxiliary gas heater set to 440 °C. The full-scan MS1 had a resolution of 35,000, with a target of 1.0 × 10^6^ and a maximum ion injection time of 100 ms. For MS2 analysis, the resolution was set at 17,500, with a maximum injection time of 60 ms, focusing on the six most abundant precursor ions from the MS1 scan. The MS2 precursor isolation window was set to 2 Da with a 0.5 Da offset. The normalized collision energy ranged from 20 to 40 eV, and unassigned ion charge states and isotope peaks were excluded from both MS1 and MS2 scans.

The LC-MS/MS data were initially in RAW format (Thermo Fisher Scientific, Waltham, MA, USA) and converted to mzML format using MSConvert 3.0.2 (ProteoWizard, Palo Alto, CA, USA).^30^ Then, the converted data were uploaded to the MassIVE repository (data set MSV000095095). MZmine 4.0.8 ^31^ was then employed to process the LC-MS/MS data. For feature detection, signal noise thresholds were set at 1.0 × 10^5^ for MS1 and 1.0 × 10^3^ for MS2 levels, respectively. The ADAP chromatogram builder created the chromatogram, with parameters including a minimum group size of 5 scans, a minimum group intensity of 1.0 × 10^5^, and a maximum intensity of 3.0 × 10^5^, with an *m*/*z* tolerance of 0.002 Da. Chromatographic deconvolution utilized the local minimum feature resolver with a minimum search range of 0.050 min and signal noise of 10 for the signal-to-noise ratio. MS1 intensity of 1.0 × 10^5^, a peak area coefficient of 1.70, and peak duration between 0.05 and 2.0 min with at least 5 scan points. Isotope detection was performed using the isotope peak grouper module, applying an *m*/*z* and RT tolerance of 0.002 ppm and 0.2 min, respectively, with a maximum charge of 2. Duplicate features were removed using the same *m*/*z* and RT tolerances of 0.30 min, with weights set at 3:1 for *m*/*z* and RT, respectively. The resulting peak list was filtered to exclude features from blanks, retaining those with isotope patterns and associated MS2 spectra. A filtered peak list containing 4472 features was ultimately exported as a .mgf file and a .csv file. Blanks were removed,^32^ resulting in a total of 4043 features for subsequent statistical analysis.

The MS feature outputs were processed using the Feature-Based Molecular Networking workflow on the GNPS2 platform (https://gnps2.org).^33^ The job can be accessed at the following link: https://gnps2.org/status?task=00dab04887e2420db77b7ff70dc48c31. The following parameters were used: precursor ion mass (MS1) and MS2 fragment ion tolerances were set at 0.005 Da each. A cosine score threshold of 0.7 and a minimum of 4 MS2 matches were required to form the molecular network and spectral searches against GNPS2 libraries.

Microbiome analyses were carried out in R Studio and metabolomics via Jupyter Notebook (all codes are available at https://github.com/pwpgomes1/Artemisia-Project). The Kruskal–Wallis test and the Wilcoxon signed-rank test were used to compare samples. Multivariate analysis was carried out utilizing the “pandas,” “sklearn.decomposition.PCA,” and “sklearn.cross_decomposition.PLSRegression” packages.

### Microbiome and Metabolomic Correlation Analysis

CLR normalized microbiome and metabolomics data were analyzed using the DIABLO approach^23^ from the MixOmics package (version 6.22.0).^34^ The DIABLO models were fine-tuned to determine the optimal number of microbiome and metabolomics characteristics to retain and the ideal number of components for fitting the data. Group-wise significance in the sparse Partial Least Squares Discriminant Analysis (sPLS-DA) along the first component was assessed using ANOVA.

## Supplementary Material



## Data Availability

The 16S rRNA sequence data is available at https://www.ncbi.nlm.nih.gov/bioproject/ under BioProject accession PRJNA1134192. Healthy stool 16S rRNA sequence data published previously may be found at NCBI under SRA accession: PRJNA497131. Metabolomics data are available at https://massive.ucsd.edu/ (data set id MSV000095095).^35^

## Notes

Overview of the integrated multi-omics analysis. This figure illustrates the experimental workflow and analysis methods used to investigate the effects of the gut microbiota collected from vegetarian or vegan subjects on crude extracts from three Artemisia species and artemisinin (commercial standard). Fecal stools from 12 healthy subjects were voluntarily collected, followed by incubation in a chemically defined medium for 48 h at 37 °C. The inoculated samples were then treated with 1% (w/v) of extracts from A. absinthium, A. californica, A. annua, such as purified artemisinin (100 μM.). Five replicates of each sample group were used for liquid chromatography-mass spectrometry and 16S rRNA analysis. Molecular networking was integrated with the DIABLO multi-omics approach to map changes in the microbiota and profile of artemisinin-related molecules. (PDF)

## ○

Prescient Metabiomics, Carlsbad, California 92008, United States
