# Supplementary figures and images for: Ex Vivo Interaction Between Human Gut Microbiota and Artemisinin: A Multi-Omics Perspective
Source: ACS Omega. 2025 May 15;10(21):21929–38. doi: 10.1021/acsomega.5c01983 (PMC12138623; doi:10.1021/acsomega.5c01983)

## SAMPLE PREPARATION

## DATA ACQUISITION

## DATA ANALYSIS

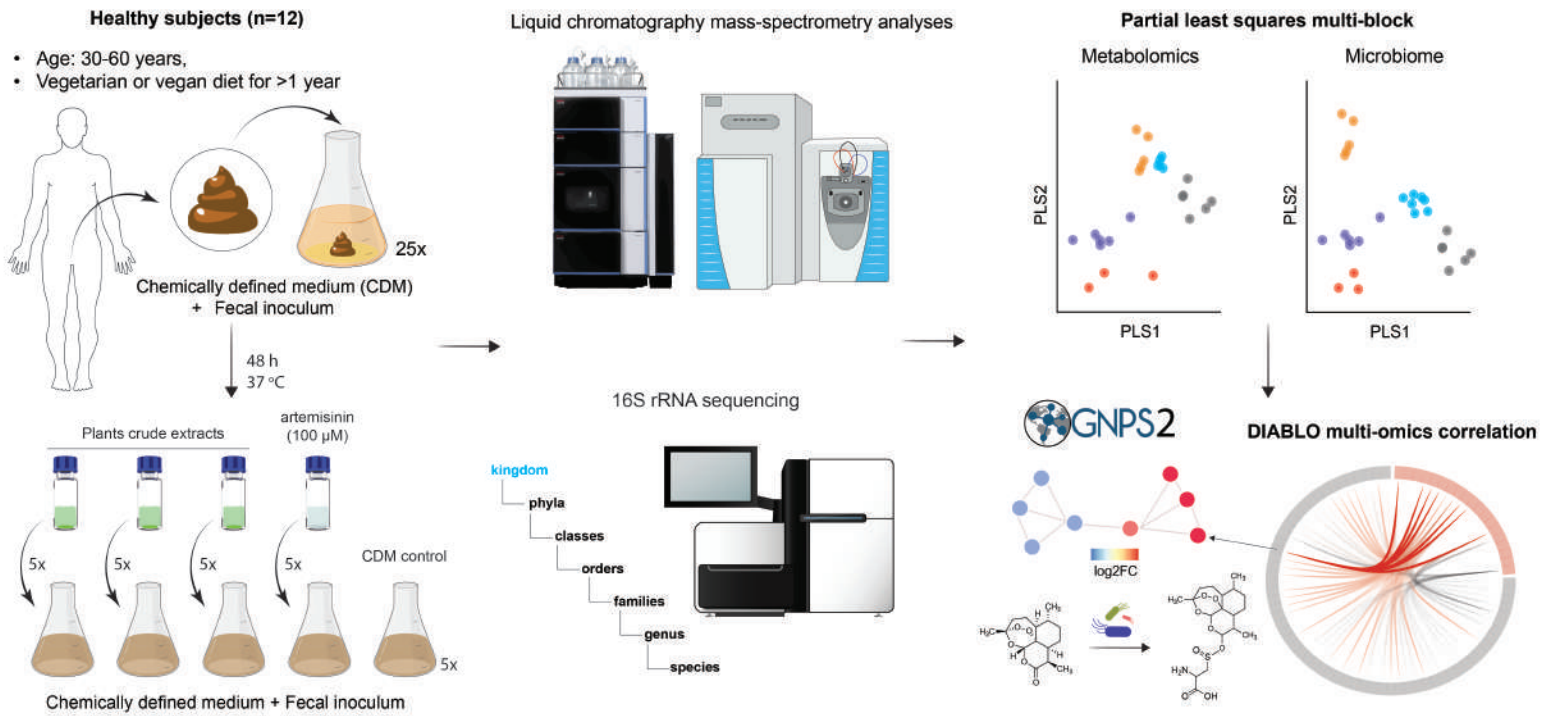

Supplement: Supplementary file 1 [file ao5c01983_si_001.pdf]
